# Sameness and the self: philosophical and psychological considerations

**DOI:** 10.3389/fpsyg.2014.00029

**Published:** 2014-01-29

**Authors:** Stanley B. Klein

**Affiliations:** Department of Psychological and Brain Sciences, University of CaliforniaSanta Barbara, CA, USA

**Keywords:** identity of self, memory, personal diachronicity, self, temporal continuity

## Abstract

In this paper I examine the concept of cross-temporal personal identity (diachronicity). This particular form of identity has vexed theorists for centuries—e.g., how can a person maintain a belief in the sameness of self over time in the face of continual psychological and physical change? I first discuss various forms of the sameness relation and the criteria that justify their application. I then examine philosophical and psychological treatments of personal diachronicity (for example, Locke's psychological connectedness theory; the role of episodic memory) and find each lacking on logical grounds, empirical grounds or both. I conclude that to achieve a successful resolution of the issue of the self as a temporal continuant we need to draw a sharp distinction between the *feeling* of the sameness of one's self and the *evidence* marshaled in support of that feeling.

Many of the constructs we grapple with in the behavioral sciences have a common-sense familiarity that makes them seem both conceptually clear and referentially transparent. However, as often is true of common-sense notions, on careful reflection they are found to be conceptually underspecified and referentially vague (e.g., Russell, 1912/1999).

The deceptively simple notion of “identity” is a case in point. When examined through an analytic lens, it becomes clear that the totality of the topics for which identity is a focal concern—e.g., personal identity, social identity, cultural identity, gender identity, national identity, object identity, numerical identity, occasional identity, contingent identity, indefinite identity, strict identity, loose identity, qualitative identity, multiple identity—is more akin to a complex fractal set (e.g., Mandelbrot, 1983) than a well-formed taxonomy. Prior to engaging questions about identity, therefore, I need to make clear how I will be using the term.

## Finding the target: what type of “identity” are we seeking?

In some contexts—primarily philosophical and mathematical—identity takes a quantitatively strict and numerically exhaustive form; it is, and only is, the realization of total property equivalence between X and Y. This is the “numerical identity” of abstract formalism, whose origins in Western thought trace to the writings of Parmenides in the 5th Century BCE (for an illuminating discussion, see Papa-Grimaldi, 2010).

But “identity” can, and often does, mean very different things in the sciences. Physical and social scientists frequently are concerned with more flexible requirements for a numerically imprecise, qualitative form of identity (e.g., specification of a subset of properties necessary and sufficient for an object to be taken as the “same” over time; this sometimes is referred to as “exact similarity,” e.g., Garrett, 1998). When questions of identity are asked of things that can take different characteristics at different times, numerical equivalence often gives way to conceptions of identity that admit to degrees and remain applicable in the presence of componential variation.

Many philosophers embrace the challenges that arise when the conditions of identity are relaxed.Numerous “puzzle cases” —amoebic cell division, the gradual replacement of an object's parts, brain transplants, split-brain surgery, body teletransportation, and many more—have received treatment (for reviews see Wiggins, 1980; Parfit, 1984; Brennan, 1988; Noonan, 1989; Oderberg, 1993; Gallios, 1998). But not all are equally accepting.

Hume, for example, felt that by allowing more flexible criteria we inadvertently substitute “similarity” for “identity” (Hume, 1739–1740/1978). Butler (1736/1819) argued that we are wrong to think that an object could gain or lose a part without bringing an end to that object: Any change in an object's constituents would, of logical necessity, bring something new into existence. On these views, we confuse identity with similarity—or, as Butler sees it, the formal notion of strict identity has been substituted by more colloquial notions of approximate or loose identity.

Substitution is not necessarily a problem, however, provided we are clear about what we are doing and our reasons for doing so. In the following section I briefly note some reasons that a change from the strict requirements of numerical identity to a more malleable conception is warranted when questions of identity are tackled by scientists. To avoid confusion, I will adopt the term “sameness” when discussing this type of less-than-perfect identity: sameness allows for less rigid, more qualitative criteria than does the quantitatively exacting demands of numerical identity (which is a type of sameness; see below).

### Degrees of sameness

In the sciences, objects of interest (whether concrete or abstract) often are held to admit to “identity” despite alterations in their properties and predicates. When entertaining the possibility of identity in the face of change, words such as “exact similarity” and “sameness” seem better suited to convey the type of identity under consideration (e.g., Williams, 1973; Noonan, 1989; Gallios, 1998; Garrett, 1998). Accordingly, rather than “identity”—which implies a binary opposition conditionalized on the presence or absence of complete property equivalence—I will use the term “sameness.” This more flexible notion allows for a spectrum of possibilities—ranging from sameness in its strict, numerical form to sameness despite (sometimes considerable) componential variation. It thus is better positioned to capture the diversity of quantitative as well as qualitative interests that characterize the numerically imprecise identities often of interest in the sciences.

In its most analytically rigid form, “sameness” entails a quantitative equivalence between X and Y. This, I suggest, typically is what we have in mind when we consider the term “identity” absent qualifying contextualization (e.g., personal identity, ethnic identity, gender identity, and so forth). Numerical sameness is a property everything has to itself and to nothing else. Formally, it is expressed as “X is the same as Y if and only if every property or characteristic true of X is true of Y as well.” Historically, this commonly is referred to as the “identity of indiscernibles,” and in modern form traces to the work of Leibnitz (e.g., Williams, 2002). Despite its inherent circularity—i.e., numerical sameness necessarily is true if and only if what is “true of X” is taken to include “being identical with X”—it remains the foundational expression of the concept of quantitative sameness (e.g., Brennan, 1988; Williams, 1990; Oderberg, 1993; Gallios, 1998). Interest in this strict version of sameness is found primarily in philosophical treatment and mathematical analysis, and will not be discussed herein.

Satisfaction of the criteria for numerical sameness seldom is in play when questions are directed toward issues of concern in the sciences^1^. Numerical sameness is an equivalence relation that must be true by virtue of the tautology it entails. Entities satisfying the requirements for numerical sameness would result in a very narrowly circumscribed set (albeit a numerically large one, since everything is quantitatively identical to itself at a given point in time). The interests of social and physical scientists more often are trained on sameness relations of entities that undergo changes wrought by the passage of time. A stone, for example, can endure erosion or supplementation (e.g., by mineral seepage), yet still be judged the same stone; a person can be considered the same person despite alterations in physical characteristics and mental states. In these domains of inquiry, equivalence, construed as numerically exhaustive, has little theoretical or empirical traction.

Accordingly, less restrictive notions are needed to accommodate the type of things toward which questions of sameness can be posed despite property variance (e.g., Brennan, 1988; Oderberg, 1993). Questions of the sameness of objects (e.g., “is that the same car I saw yesterday?”), propositions (e.g., “on closer analysis, the two theories seem to be the same”), mental states (e.g., “I think we have the same idea”) and more complex cases (e.g., the self: “Am I the same person I was 10 years earlier?”) allow for the possibility that X and Y are, in some sense, the same despite not satisfying the strict requirements for numerical equivalence. An important consequence of this relaxation in criteria is that it draws greater attention to the thing being evaluated, broadening the scope of analysis to include consideration not only of the sameness relation, but also of the nature of the relata placed in relation. This change in accent, as we will see, takes a particular significance when the object of inquiry is one's self (e.g., Shoemaker, 1963; Wiggins, 1971; Rorty, 1976; Hirsch, 1982; Baillie, 1993; Garrett, 1998; Baker, 2000; Lund, 2004; Perry, 2008; Sani, 2008)^2^.

## Sameness and the self: the problem of personal diachronicity

As Hume's and Butler's insights suggest, when addressing questions of identity in the sciences we often loosen the requirements for numerical sameness. Our concern is how people judge or perceive the qualitative sameness of an entity despite changes with time. When the person is taken as the object of a sameness judgment, the requirements of quantitative equivalence would, of definitional necessity, preclude affirmation for any observation falling outside the narrow boarders of instantaneity: the continual change associated with the psycho-physical existence would make personal diachronicity (i.e., the sameness of the person over time) a logical impossibility (unless one subscribed to a view in which change is an illusion, and the reality behind the illusion is in a state of stasis; for discussion see Barbour, 2000 and Papa-Grimaldi, 2010).

Quantitative sameness clearly is *not* what we have in mind when the sameness of persons is in question; rather, we are interested in criteria that can be used to justify a belief that Person X is the same at time *T*_1_ and at time *T*_2_. Under these circumstances, conditions satisfying the tautological certainty of numerical sameness give way to the search for criteria capable of allowing for the possibility personal sameness despite alteration in properties or predicates. As we will see in the section titled Types of Self and Types of Personal Diachronicity: Evidence and Certainty, criterial emendations are particularly complex when the object of a sameness judgment also is the one making the judgment—i.e., the sameness of one's self.

Accordingly, questions of strict, numerical identity are not (and cannot be) the concern theorists interested in personal diachronicity (adoption of such criteria would result in an empty set). Rather, our interest is in the criteria we rely on to attribute spatio-temporal *continuity* to persons in general and the self in particular. And, of logical and empirical necessity, these criteria must entail the flexibility necessary to ascertain sameness despite inevitable transformations in a person's physical and mental constituents.

In short, despite the use of the word “same” to categorize our theoretical and empirical interests in the sameness of self (e.g., personal identity), we are concerned not with sameness in its strict, Liebnizian sense, but rather with a more qualitative question of personal sameness or diachronicity. Indeed, when taken as numerical equivalence, the question of personal sameness has no meaning (save for the possibility of a Parmenidean vision of reality as static perfection; e.g., Papa-Grimaldi, 2010).

Personal diachronicity (which henceforth will be restricted in application to the “self”) is unique among topics amenable to considerations of sameness. In addition to judgments made by the self-as-subject of the self-as-object (see the next section), sameness pertains also to judgments by the self-as-subject of the self-as-subject. These self-reflexive^3^ acts are limited to sentient beings and likely apply with reasonable assurance only to homo sapiens (e.g., Snodgrass and Thompson, 1997; Terrace and Metcalfe, 2005). However, before pursuing the conditions that must be satisfied to justify a judgment of personal diachronicity, we need to make explicit what it is we take to be the target of the sameness judgment—the self.

### The problem of the self

As those who study the self-have discovered, answers to the question “What *is* the self?” are elusive at best (for reviews see Johnstone, 1970; Gergen, 1971; Lewis, 1982; Vierkant, 2003; Klein, 2012). Indeed, some are of the opinion that the question is based on the illusion that there is an elusive self to be found (e.g., Albahari, 2006; Metzinger, 2009; for discussion see Siderits et al., 2011). Of course, a problem with this perspective is that an illusion is an experience and an experience requires an experiencer (e.g., Strawson, 2011a; Klein, 2014a). As Meixner (2008) observes, “The fictionalization of subjects of experience is incoherent, since it involves the incoherent idea that I, for example, am an illusion of myself” (p. 162). Kant (1998) goes further, arguing that the self of subjective awareness (his transcendental ego) must accompany experience” [related views can be found in James (1890), Lund (2005)].

Despite ontological concerns, psychology has found work for the “self” in an abundance of subject-hyphen-predicate relations (e.g., self-comparison, self-concept, self-esteem, self-handicapping, self-image, self-perception, self-regulation, self-reference, etc.). However, the focus of investigation rests firmly on the predicate, to the detriment of an appreciation of what exactly is the object of this diverse set of predicates—i.e., the self being verified, conceptualized, esteemed, deceived, verified, regulated, and handicapped (for review see Klein, 2012, 2014a).

This is not to say that psychology has failed to propose models of the self: formalizations have been on display for more than 100 years [e.g., James, 1890; Greenwald, 1981; Neisser, 1988; Kihlstrom and Klein, 1994; Conway, 2005; for recent reviews see Leary and Tangney (2012) and Sedikides and Spencer (2007)]. Yet, most of these offerings target the self in a particular context, rather than the self *per se*. We thus find models of cultural selves, social selves, cognitive selves, synaptic selves, autobiographical selves, social selves, narrative selves, etc. (cf., Leary and Tangney, 2003, 2012). But consideration of what the self *is* that serves as the bedrock of these cultural, social, cognitive, synaptic, and narrative instantiations, typically is under-specified (e.g., Klein and Gangi, 2010; Klein, 2014a).

### The two selves: the neural self of science and the subjective self of first-person phenomenology

One reason for the difficulties we face when attempting to describe what we mean by the word “self” is that there is not a single self to be described (e.g., Stern, 1985; Neisser, 1988; Klein, 2001, 2004, 2012, 2014a; Legrand and Ruby, 2009). Rather, two distinct (but normally interacting) aspects of the self are conjoined in almost every discussion of the topic, although these aspects seldom are separated. As reviewed at length in Klein (2012, 2014a), the self meaningfully can be partitioned into the neurally instantiated systems of self-knowledge and the self of first-person subjectivity (e.g., James, 1890; Zahavi, 2005; Legrand and Ruby, 2009; Strawson, 2009; Klein, 2012, 2014a).

It is beyond the scope of this paper to go into detail about the material and subjective aspects of self [extensive discussion can be found in Klein (2012, 2014a)]^4^. Briefly, they cannot be deduced from, or reduced to, a single, underlying principle, structure, process, substance or system (e.g., Kant, 1998; Zahavi, 2005; Klein, 2012, 2014a). One—the neuro-cognitive systems of the psycho-physical self (consisting of such things as personal memory, body image, emotions)—is materially (primarily, but not exclusively, neural) instantiated and therefore capable of being apprehended and treated as an *object* of scientific inquiry.

The other—the self of first-person subjectivity—is the *subject* having the experience, rather than the *object* of that experience. This aspect of self cannot be directly known by acts of perception or introspection (e.g., Earle, 1972; Kant, 1998; Zahavi, 2003, 2005; Lund, 2005; Klein, 2012; Swinburne, 2013). Rather, our appreciation of the self of first-person subjectivity is a matter of acquaintance or feeling, something that cannot (easily) be conveyed via descriptive analysis (e.g., Nagel, 1974; Kant, 1998; Zahavi, 2005; Klein, 2012, 2014a).

Despite differences in their epistemological (and possibly ontological; e.g., Klein, 2014a) status, under normal circumstances these two aspects of self-interact, and this interaction is a prerequisite for our experience of self. Indeed, it is *only* via their interaction that a particular form of consciousness—self-awareness—becomes possible [these assertions are treated extensively in Klein (2012, 2014a); see also Gallagher and Zahavi (2008)]. In this regard, I follow Fitche's dictum (e.g., Neuhouser, 1990) that there can be no subject without an object or object without a subject.

Considerable progress has been made describing the cognitive and neurological bases of the material aspects of self (recent treatments can be found in Conway, 2005; Klein and Gangi, 2010; Klein and Lax, 2010; Renoult et al., 2012; Martinelli et al., 2013; Prebble et al., 2013). This is because the material, neuro-cognitive bases of self-knowledge can be (and have been) objectified, and thus amenable to scientific analysis.

The subjective aspect of self, by contrast, is too poorly understood to bear the definitional weight required when placed in relation to predicates (e.g., regulation, image, complexity, handicapping, verification, etc.) or contexts (e.g., synaptic, cultural, narrative, etc.). Moreover, as discussed below, treating the subjective self as an object has the unfortunate consequence of stripping it of its core feature—its subjectivity (for discussions see Zahavi, 2005; Ganeri, 2012; Klein, 2012, 2014a).

Researchers often fail to appreciate that the self of first-person subjectivity is *not* the object of their experimental inquiries (e.g., Klein, 2012; Klein and Nelson, 2014). Nor could it be. Objectivity is based on the assumption that an event or object exists independent of any individual's awareness of it (e.g., Earle, 1955; Nagel, 1974; Rescher, 1997; Martin, 2008); it is something other than self. When objectivity is the stance adopted by the self to study itself, the self must, of logical necessity, be directed toward what is not self—i.e., to some “other” that serves as the self's object (e.g., Husserl, 1964; Earle, 1972; Lund, 2005; Zahavi, 2005; Klein, 2014a). Thus, to study myself as an object, I must transform myself into an “other,” that is, into a “not-self,”

Accordingly, the subjective self is not, and cannot, be an object for itself and still maintain its subjectivity. Considered by first-person subjectivity, the subjective aspect of self becomes an object in the manner all objects (both mental and physical) must, of necessity, become when apprehended (e.g., Husserl, 1964; Zahavi, 2003; Klein, 2012). In the process, the subjective aspect of the self of first-person experience is lost from view. Paradoxically, the subjective aspect of self can achieve objectivity only at the cost of forfeiting its essence as a subjective center (e.g., Kant, 1998; Zahavi, 2005; Klein, 2012, 2014a).

## Types of self and types of personal diachronicity: evidence and feeling

Personal diachronicity concerns our belief that we have an identity that originated in our past and will follow us into our future. Although most treatments take this to be a question of how we *know* (I am using “knowledge” in its non-technical, colloquial sense, rather than its philosophical sense as true, justified, belief) that we are the same over time, a second, equally important aspect of diachronicity often is overlooked—i.e., on what do we base our *feeling* that we are continuous in both temporal directions from the present?

Different criteria come into play depending on whether the self-posses “itself to itself” as an object or as a subject. When treated as the object of subjectivity, criteria that enable us to know that we are the same despite componential change are relevant. I refer to these knowledge-based criteria as *evidential* sameness.

When the self as subject takes its own subjectivity as the basis for sameness, by contrast, the criteria for sameness are felt. In contradistinction to evidential criteria—i.e., a consideration of facts relevant to a diachronicity judgment and the inferences such considerations permit—one's *feeling* of sameness derives from one's pre-reflective feeling that despite change in the object of awareness, the subjective “I” by which the object is apprehended remains unchanged (the potentially ageless nature of the subjective self is addressed in the section titled The Timelessness of the Subjective Self). The feeling of the sameness of the subjective self is a-theoretic—it is feeling devoid of reason and directly apprehended (for discussion, see Earle, 1955; Kant, 1998; Zahavi, 2005; Gallagher and Zahavi, 2008; Klein, 2012, 2014a).

Thus, when questions of sameness are addressed to the self, the answers we seek depend in important ways on the aspect of self being judged. To anticipate my conclusions, logically viable and empirically justifiable arguments for the continuity of the material aspects of self—both its physical properties and psychological features clearly tied by experimental evidence to neural activity (e.g., memory, perception, and so on) are hard to come by: there simply are no unambiguous *evidential* criteria (at least, given the resources currently on hand) capable of underwriting a belief in the diachronicity of the material aspects of self.

In contrast, one's *sense* of personal diachronicity is sustainable when the aspect of self under consideration is its subjectivity. But this comes at a cost—our criteria for sameness, being felt rather than known, are not amenable (in any obvious way) to quantitative, evidential analysis (for discussion, see the section titled The Problem of the Self). And this may seem too high a price to those entrenched in a materialist world view (for discussion, see Papa-Grimaldi, 1998; Meixner, 2005; Koons and Bealer, 2010; Nagel, 2012; Klein, 2014a).

## Philosophical treatments of personal diachronicity: evidential sameness and the material self

Let's begin by examining the evidential criteria commonly used to address the sameness of the material self. Questions of the sameness of the material aspects of self can ordered roughly with regard to their scope or inclusiveness. At the most general level, questions of the sameness can be posed to the self qua physical body: What is the relation between bodily continuity and personal diachronicity?

While Bodily Criteria have been subject to extensive philosophical analysis and debate (e.g., Williams, 1973; Parfit, 1984; Olson, 1997, 2007; Baker, 2000), on examination it becomes apparent that not all parts of the body carry equal evidential weight. One organ—the brain—seems particularly germane to evidence-based treatments of personal diachronicity.

However, even at this more nuanced level, cracks in the criterial base begin to appear. Ultimately, we find that the criteria for continuity of the material aspects of self, if they are to have any possibility of evidential warrant, must focus on its psychological, rather than its physical properties (e.g., Parfit, 1984). I refer to these as Informational Criteria. One psychological property in particular—the continuity of personal memory—traditionally has been taken by psychologists and philosophers alike as the *most likely* informational candidate for grounding judgments of personal diachronicity in an evidential nexus (for reviews see Perry, 2008; Sani, 2008). In this paper, I restrict analysis largely to this aspect of the material self; I only briefly mention a few of the less well-studied informational candidates - e.g., empathic access).

### The bodily criterion

The most general level at which evidence for personal diachronicity might be found comes from analysis of the conditions required to bestow spatio-temporal continuity on the body. As is the case for all physical objects, rapidity of change plays a critical role for judgments of diachronicity (e.g., Spencer Brown, 1957; Campbell, 2004). This is made salient by consideration of Plutach's famous paradox of the “Ship of Theseus” (for discussions see Wiggins, 1980; Brennan, 1988; Noonan, 1989; Oderberg, 1993). In one of its several adaptations (the one most relevant to personal diachronicity), the question posed is whether a ship which has had some or all its planks replaced remains the same ship?

Variations in the replacement schedule play a critical role in the answers one is likely to intuit (e.g., Campbell, 2004). Gradual replacement of the ship's planks (e.g., one at a time, at a leisurely pace), generally support the inference that the ship remains the same. If change is too rapid, however, one's certainty of the ship's continuity is challenged. Yet it is important to bear in mind that all that varies between scenarios is the rapidity of change, not change itself. Differences in sameness judgments appear to trade, to some degree, on temporal considerations.

Judgments of the sameness of objects also pivot on amount of change. Most of us are willing to grant sameness to a ship that has one, or a few, planks replaced. But judgment is less secure when the ship undergoes substantial (or complete) physical alteration, even when the change is gradual. Some have proposed quantitative boundaries beyond which confidence in sameness drops precipitously (for example, Parfit, 1984, suggests componential replacement exceeding 50% has serious negative consequences for sameness judgments). But these numerical constraints are based more on reasonable intuition than on logical analysis or experimental demonstration.

When the sameness of the material self is called into question, a similar set of issues arise. We constantly are adding to and subtracting from our body—e.g., as we age we grow taller, gain and lose pounds, change cells, molecules and atoms. The degree of bodily change can be extraordinary: By some accounts all the atoms in our body are replaced over a 10 year span. The mental properties of the material self-change as well—e.g., we gain and lose knowledge, add and lose memories, acquire new skills, modify goals, and so on.

If change (whether physical or mental) happens slowly, most of us assume we are the same person today we were 1 min, 1 h, or one decade earlier (e.g., James, 1890; Hirsch, 1982; Brennan, 1988; Campbell, 2004)^5^. But is this belief justified (e.g., Wiggins, 1971, 1980; Oderberg, 1993)? If, as per impossible, the “me” of age 60 were to meet the “me” of age 10, most of the evidential bases for spatio-temporal continuity clearly would be lost. The old “me” would bear neither a physical resemblance to the young me, nor would we share many experiences, beliefs, goals, memories and other mental features. In short, these temporally separated, gradually altered selves would have little in common—save a largely intact genetic code. Should we meet, we likely would meet as strangers (although the older “me” might “know better”). In what would our sameness consist?

### The brain criterion

As many philosophers have observed, not all aspects of the body are equally positioned to underwrite personal diachronicity (e.g., Shoemaker, 1963; Williams, 1970; Wiggins, 1971; Noonan, 1983; Baker, 2000; Olson, 2007). One part of the body in particular—the brain—seems disproportionately relevant to questions of the sameness of the material self. Perhaps by focusing on a more restricted range of bodily parts, some of the problems associated with the Bodily Criterion can be avoided.

The role of the brain in determination of personal diachronicity is placed in sharp relief by a thought experiment, popularized by Shoemaker (1963) and subsequently elaborated on by Parfit (1984). In the original scenario, Mr. Brown has his brain transplanted into Mr. Robinson's body. Let's call the resulting individual—consisting in Robinson's body and Brown's brain—Mr. Brownson. Assuming the operation was successful, “what is the identity of Mr. Brownson?”

When philosophers (and non-philosophers alike) are asked to reflect on this scenario, the common intuition is that Brownson is the same as the original Mr. Brown (e.g., Noonan, 1989). This suggests that a broad Bodily Criterion must give way more circumscribed view in which certain body parts count more than do others in determinations of personal sameness. What appears required for continuity of the self is not the body, taken en toto, but rather one of its parts—the brain.

### The informational criterion

However, even the Brian Criterion may be too gross a characterization of what matters for personal diachronicity (e.g., Proust, 2003). The brain, after all, simply is the part of the body that happens to host memory, personality, mood, thought and a number of other psychological faculties and functions. Perhaps body-based criteria for the re-identification of the material self, even those restricted to the brain, are not the best place to search for evidentially-based criteria for self-continuity.

The argument can (and has been) made that what serves as the criterion of sameness is not the persistence of the physical brain, but rather the continuity of the personally-relevant *information* contained within that body part. Although this information contingently is located in the brain, the continuity of the information, not of the organ in which it is housed, is what really matters (e.g., Williams, 1973; Parfit, 1984; Brennan, 1988; Noonan, 1989; Gallios, 1998).

Consider, as an example, the case of information transfer popularized by Parfit (1984). Imagine there is a machine capable of extracting all the information in Person X's brain and transferring it to the brain of Person Y, and vice versa. Under this “science-fiction” scenario, who would be Person X and who would be Person Y? As Williams (1970) and many others (e.g., Wiggins, 1971; Noonan, 1989; Baillie, 1993; Garrett, 1998) see it, the answer is clear—where knowledge goes identity follows.

The most famous version of the Information Criterion is contained in a passage from Locke: “Personal identity—that is, the sameness of a rational being—consists in consciousness alone, and as far as this consciousness can be extended backwards to any past action or thought, so far reaches the identity of that person.” (Locke, 1689 Bk. II, Ch. 27, Sec. 9). Although, as we will see in the section titled The Need to Take Seriously The Self of First-Person Subjectivity in Accounts of Personal Diachronicity, there is some question about exactly what Locke had in mind here (e.g., Strawson, 2011b), the passage usually is taken to involve a person remembering self-referential action or thought (e.g., Shoemaker and Swinburne, 1984; Noonan, 1989), that is what cognitive psychologists call episodic memory (e.g., Tulving, 1983).

Building on this reading, a prominent interpretation of Locke's view goes as follows: A person at one time, *P*_2_ at *T*_2_, is the same person at an earlier time, *P*_1_ at *T*_1_, if and only if *P*_2_ can remember having done and experienced various things performed by *P*_1_ (e.g., Shoemaker, 1963; Greenwood, 1967; Noonan, 1989; Schechtman, 1990; Proust, 2003). Thus, it is the transitivity of episodic memory that establishes the continuity of self.

Similar views are common in psychology (for reviews see Fivush and Haden, 2003; Sani, 2008). An especially clear exposition is offered by two prominent neuroscientists: “We are not who we are simply because we think. We are who we are because we can remember what we have thought about…. Memory is the glue that binds our mental life, the scaffolding that holds our personal history and that makes it possible to grow and change throughout life. When memory is lost, as in Alzheimer's disease, we lose the ability to recreate our past, and as a result, we lose our connection with ourselves and with others.” (Squire and Kandel, 1999, p. ix). On this analysis, what makes a person the same across time are relations of memory: it is by memory of past action that the self attains a sense of continuity.

Because Locke's memory-based account (and by memory he typically is taken to mean episodic memory) has received the bulk of attention from philosophers and psychologists, I focus on this aspect of the informational criterion in what follows. However, the reader should be made aware that this is not the only candidate for an informational criterion capable of supporting our belief in personal diachronicity (I briefly mention a few others, though my treatment rests firmly on the evidential offerings of memory).

Unfortunately, as his critics were quick to note, Locke's account seems to entail a vicious form of circularity (Butler, 1736/1819; Reid, 1813/1969). For a mental state to count as my memory of a past action, it has to be the case that I was the one who performed the past action. If it wasn't me who performed the action, then my apparent recollection is simply a mistake, not a memory. Butler states the problem bluntly: “one should really think it self-evident, consciousness of personal identity presupposes, and therefore cannot constitute, personal identity” (p. 290). If memory presupposes sameness of self, then trying to give an account of identity in terms of memory seems hopeless.

Although the circularity objection is a serious problem for any simple version of Lockean theory (e.g., Williams, 1973; Brennan, 1985; Noonan, 1989; Proust, 2003), many still favor a memory-based account of personal diachronicity (as opposed to, say, a bodily account; see Olson, 2007). Accordingly, a number of emendations have been proposed to rein in the tautology (e.g., Schechtman, 1990; Hamilton, 1995; Collins, 1997; Slors, 2001; Klein and Nichols, 2012; for review, see Bernecker, 2010).

I have discussed the circularity objection at length elsewhere, and presented evidence that episodic recollection and the self are contingently, not logically, intertwined (Klein and Nichols, 2012). Treatment of these issues would take us far beyond the scope of the present paper. Instead, I focus on the other well-known criticism of Locke's memory criterion—i.e., that it cannot work due to “gaps” that necessarily occur in our memorial record. This issue of episodic transitivity has exercised theorists from the earliest days of the debate.

Hume (1739–1740/1978), conceptualizing the problem in terms of numerical sameness, asks how could there be a quantitatively strict sameness across time in light of the fact that a person's psychology constantly is changing? Reid (1813/1969) also takes issue with Locke's memory criteria, arguing that even less numerically exacting accounts present seemingly insurmountable difficulties (although he famously rejects the memory theory of personal identity, Reid does acknowledge that memory seems to provide “irresistible” evidence that I am the very person who did the action; 1813/1969).

Suppose, Reid observes, a military officer had been flogged for robbing an orchard when he was a boy at school, had bravely vanquished an enemy during battle, and had been made a general later in life. Further, suppose that when he won his military campaign, he could remember having been flogged at school and that when made a general he was remembered his military victory but no longer remembered his flogging.

As Reid sees it, if a person at time *t_n_* remembers an event that occurred at time *t*_1_, then the person at time *t_n_* is identical with the person who was witness to or the agent responsible for the event at time *t*_1_. Thus, if the brave officer who defeated the enemy remembers being beaten at school, then the officer is identical with the boy who was beaten. By similar logic, if the general remembers defeating the enemy in battle, then the general is identical with the brave officer. If the general is identical with the brave officer, and the officer is identical with the boy, then, by the logic of transitivity, the general is identical with the boy.

However, since the sameness of memory is a necessary condition for sameness of self, if a person at time *t_n_* does not remember an event that occurred at time *t*_1_, then the person at time *t_n_* cannot be the same as any person who was witness to or agent of the event at time *t*_1_. Thus, if the general cannot remember being beaten at school, he cannot be the same as the boy who was beaten. Locke's memory account thus suffers from a set of mutually incompatible theses—i.e., the general is both the same as and different from the boy.

Williams (1973) has identified another obstacle facing an evidential account of personal sameness based on memory criteria. He invites us to imagine a situation in which the memory claims of Person X are continuous with those of deceased Person Y. That is, Person X's memory claims map unanimously with the life-history of Person Y. Does this mean that Person X is Person Y? And if so, does this mean that a person can be alive and dead at the same time? It is clear that memory-based evidence (and episodic recollection in particular) suffers from problems that render the utility of the Informational Criterion less than optimal.

Some have attempted to circumvent these problems by proposing that information other than memory might provide the evidential basis for judgments of personal diachroncity. Schechtman (2001), for example, suggests we shift emphasis from an exclusive reliance on memorial criteria to what she calls “empathic access”—i.e., one's psychological make-up, broadly construed to include desires, feelings, goals, values, beliefs, memory, etc. (Schechtman is not alone in this regard, though others do not adopt her terminology of “empathic access”). Others have argued that relaxing the requirement of immediate access to a temporally continuous succession of remembered events might avoid the problem of “gapy” memorial records (e.g., Brennan, 1985). On this account, it is sufficient that we show enough coherence in our recollections to merit the assignment of sameness to a person.

But, with regard to the former approach, potential gaps and issues of transitivity still remain in play even when mental states other than those strictly taken as memory are recruited as evidential criteria (for discussion, see Klein, 2014b). And the relaxation argument is shown to be inadequate in light of circumstances in which individuals maintain a sense of personal continuity despite the *complete* loss of episodic memory (as we will see in the next section). In addition, it is unclear just what constitutes “enough” coherence.

## Psychological treatments of personal diachronicity: evidential sameness and the material self

Most philosophical treatments of personal diachronicity, as we have seen, rely on “thought experiments” to identify the evidential bases of sameness judgments. Arguments resulting from this “mental empiricism” are believed viable if they can be shown to be internally consistent and logically coherent.

Recently, however, philosophers have begun to question the utility of thought experiments unconstrained by scientific empiricism (e.g., Wilkes, 1988; Focquaert, 2003). Coherence and consistency may allow us to judge the logical warrant of a criterion, but conceivability should not be confused with empirical possibility. Perhaps if logical considerations were supplemented with empirical evidence, there still might be hope for a memory-based approach to personal sameness.

Psychologists apparently think so: Many accept (often uncritically) the idea that memory—in particular, its episodic component —is the basis of personal diachronicity (e.g., Rubin, 1986; Conway, 2005; Markowitsch and Staniliou, 2011; Bluck and Liao, 2013; for reviews see Fivush and Haden, 2003 and Sani, 2008). Neurological case studies appear especially suited to shedding light on this issue (e.g., Rathbone et al., 2009; Illman et al., 2011; Duval et al., 2012; Picard et al., 2013; Klein, 2014b). Specifically, cases of neurological impairment offer the possibility of observing dissociations between a belief in one's temporal continuity and the neurological mechanisms posited to support that belief. In this way, one can examine the extent to which belief in the sameness of self contingently depends on the availability of neurally instantiated informational criteria.

When examined critically, however, the evidence is not encouraging. As I show below, episodic memory cannot, by itself, do the work needed to underpin one's belief in one's sameness over time. While recollection may be *useful* in response to personally or socially motivated requests for evidential support, case studies have shown that episodic memory can be lost (even completely) without any obvious consequences for one's sense of diachronicity (for reviews see Klein and Gangi, 2010; Craver, 2012; Klein, 2012, 2014b). In short, empirical evidence (as well as logical considerations; e.g., the issues of non-transitivity identified by Reid) make clear that while episodic memory may be sufficient for one's sense of personal continuity, it is not necessary^6^.

### Semantic memory and personal diachronicity

Before abandoning a memorial criterion, however, it is important to keep in mind that the self is represented in systems other than episodic memory (for reviews, see Klein, 2004; Gillihan and Farah, 2005; Klein and Gangi, 2010; Klein and Lax, 2010; Renoult et al., 2012; Martinelli et al., 2013). Within semantic memory, for example, there are (at least) two different subsystems devoted to autobiographical knowledge (for review and discussion, see Klein and Lax, 2010). One contains factual self -knowledge (e.g., “I am 61” and “I live in Goleta”). The other is the repository of knowledge of one's personality traits (e.g., “I am intelligent” and “I am not punctual”).

There now exists an extensive data-base showing that patients suffering episodic amnesia still can retain access personal facts and trait characteristics (for evidence and reviews see Tulving et al., 1988; Tulving, 1993; Klein et al., 1996; Rathbone et al., 2009; Klein and Gangi, 2010; Klein and Lax, 2010; Martinelli et al., 2013). It is possible, some have suggested, that one's sense of personal identity can be maintained by semantic forms of self-knowledge (factual and trait) in the presence of episodic amnesia. Consistent with this position, evidence suggests that one's sense of personal sameness is not lost despite (sometimes pervasive) episodic memory impairment (e.g., Rathbone et al., 2009; Haslam et al., 2010; Illman et al., 2011; Duval et al., 2012; Klein, 2014b).

### A dissociation between factual self-knowledge and trait self-knowledge

These cases and others like them [reviewed in Klein and Lax (2010)] demonstrate a dissociation between episodic and semantic forms of self-knowledge^7^. But can semantic knowledge of one's traits dissociate from other types of semantic knowledge (both self- and non-self-referential)? Further testing suggests that it can.

Consider the case of Patient D.B., a 79-year old man who became profoundly amnesic as a result of anoxia following cardiac arrest. One particularly noxious consequence of his anoxia was that it rendered him incapable of episodically recollecting a *single* thing he ever had done or experienced.

To test his semantic trait self-knowledge, we asked D.B. on two separate occasions to judge a list of personality traits for self-descriptiveness. We also asked his 49-year-old daughter (with whom he lives) to rate him on the same traits. Our findings revealed that D.B.'s trait ratings were both reliable and consistent with the way he is perceived by others (for analyses and discussion see Klein et al., 2002c). Moreover, his access to trait self-knowledge was indistinguishable from age-matched, neurological healthy controls. He thus maintained accurate and reliable knowledge of his personality despite lacking access to specific actions and experiences on which that trait knowledge was based. A similar picture is presented by patient K.C. Tulving (1993). Despite suffering a complete loss of episodic memory, K.C.'s ability to access trait self-knowledge remained intact.

Although D.B. knew which traits described him, he had considerable difficulty accessing semantic-based factual self-knowledge. For example, he no longer could recall the names of any friends from his childhood or even the year of his birth. He also showed spotty knowledge of facts in the public domain. For instance, although he was able to accurately recount a number of details about certain historical events, his knowledge of other historical facts was seriously compromised (e.g., he claimed that America was discovered by the British in 1812).

Taken together, these findings evidence dissociations *within* semantic memory. On the one hand, D.B.'s general semantic knowledge and factual self-knowledge was impaired; on the other hand, his semantic trait self-knowledge was spared and, at least with respect to the measures used, indistinguishable from that of control participants.

Moreover, his ability to retrieve trait self-knowledge was not due simply to the sparing of the systems responsible for maintaining a data-base of trait knowledge (whether about self or other). For example, D.B. was unable to produce accurate knowledge of his daughter's traits (e.g., Klein et al., 2002c). Similar selectivity favoring trait self-knowledge also has been found to characterize autistic memory function (e.g., Klein et al., 1999, 2004).

These findings suggest that the resilience of trait self-knowledge is not a general property of semantic trait-knowledge. Rather, it appears specific to trait generalizations about the self. Indeed, my colleagues and I have yet to find a population (e.g., amnesia, autism, ADHD, Alzheimer's Dementia, Prosopagnosia, Schizophrenia) that cannot reliably and accurately report knowledge of their own traits despite (often considerable) disruption of other neurological and cognitive function (for reviews see Klein and Lax, 2010; Klein et al., 2013).

In contrast to the conclusions just voiced, work reported in a volume edited by Prigatano and Schacter (1991) suggests that people suffering deficits following neural injury sometimes do not recognize the extent to which particular trait-based characterizations apply to them. In addition, evidence is presented that patient and family members may give different answers to questions about traits that describe the patient.

However, as discussed at some length in Klein et al. (2013), the question of the stability of one's beliefs about his or her personality traits does not trade on agreement between one's views and those of others. People—whether brain damaged or fully intact—often disagree with others about which traits best describe them (e.g., Klein et al., 2002a). The question is whether a person's beliefs about his or her dispositions remains stable (even if at odds with the beliefs of others) over time, not the assumed accuracy of those beliefs. And with respect to the former concern, the evidence is that our beliefs about our dispositions remain remarkably stable even in the presence of considerable neurological damage and cognitive chaos.

Returning to the question of personal diachronicity, a review of the evidence suggests that that individuals suffering loss of both episodic *and* factual semantic knowledge still have a sense of temporal self-extension (Klein, 2012, 2014b). Perhaps, then, the remarkable stability of semantic trait self-knowledge provides the bedrock from which one's sense of personal diachronicity springs.

In summary, with respect to the Evidential Criterion, long-term memory does not seem necessary for one's feeling of personal identity across time (for a similar conclusion, see Craver, 2012). The fact that patients like D.B. lack access to episodic memory and show impairments of factual semantic personal memory yet maintain a sense of personal diachronicity (possibly influenced to some degree and in some, as yet, unspecified manner by the stability of semantic trait self-knowledge) suggests one does not need either episodic memory or factual semantic self-knowledge to experience the sameness of self [a similar conclusion, based on philosophical considerations, is found in Strawson (2005)].

## Personal diachronicity and the subjective self

“If we would have true knowledge of anything, we must quit the body.”

(Phaedo, quoted in Russell, 1949, p. 159).

Thus, far I have examined some of the evidential criteria by which we might make judgments of personal sameness over time. These criteria apply in their most straight-forward manner to those aspects of the self that fall under the heading “material”—i.e., the psycho-physical features of self-amenable to objectification. Unfortunately, as we have seen, with the possible exception of trait self-knowledge, the utility of this evidence for underwriting our sense of personal continuity is at best questionable.

There is, however, another aspect of self—its first-person subjectivity—that has received little attention as a possible basis of personal diachronicity. Is there any reason to suspect this aspect of self may serve as the foundation of our feeling of diachronicity? I believe there is, and my reasons for so believing, as well as the empiricism on which they are based, are the focus of the next several sections of this paper.

### The need to take seriously the self of first-person subjectivity in accounts of personal diachronicity

First-person subjectivity is a universal aspect of our experience of self; one that, despite well-known difficulties situating it in a materialist framework (e.g., Klein, 2014a), is a phenomenological reality that cannot be ignored if one is to fully appreciate what it means to be a self (e.g., James, 1890; Kant, 1998; Lund, 2005; Zahavi, 2005; Dainton, 2008; Legrand and Ruby, 2009; Strawson, 2009; Klein, 2012, 2014a). Equations and measurements can be useful when they are related to experience; but experience comes first (e.g., Gallagher and Zahavi, 2008; Klein, 2014a).

It is undeniable that many, if not all, of the great achievements in modern science were made possible by the exclusion of “subjectivity” from the world around us. However, a comprehensive appreciation of reality must include that aspect of reality that makes its understanding possible—i.e., the subjectivity of self that provides us with the ability to be aware of the world of which it is a part. To do otherwise is to exclude by stipulation that aspect of nature that makes nature knowable to itself. As Ricard and Thuan (2001) observe, “If we define the terrain field of science as what can be physically studied, measured, and calculated, then right from the start we leave out everything that is experienced in the first person, and all immaterial phenomena. If we forget this limitation, then we soon start affirming that the universe is everything that can be objectified in the third person, and only what is material.” (p. 241).

It thus seems prudent to consider the possibility that our sense of self-sameness derives from feelings obtained from and apprehended by the conscious aspect of self. In this regard, it is interesting to note that Strawson (2011b) has made a strong case for taking Locke at his word—to wit, when Locke posits the continuity of consciousness as the foundation of diachronic personal identity, he means just that: Continuity derives from the felt invariance of subjectivity (i.e., the subjective aspect of self), *not* from evidential (e.g., memorial) sources which subjectivity takes as its objects (which are in a continual state of change). On awakening each morning, I immediately am aware of my self, that “I” exist. My feeling of self as a psychological continuant is not something I need to deduce or reconstruct to justify my feeling of continuity^8^. As Heidegger observes, “I am always somehow acquainted with myself” (1993, p. 251). Locke is more blunt: “consciousness *alone* makes self” (Locke, 1689 Bk. II, Ch. 27, Sec. 9; emphasis added).

While non-memory impaired individuals can recollect material with self-referential content—and often do so for legal, personal, or, more typically, social reasons—such recollections do not appear to be *required* for one's *feeling* of personal continuity. During most waking moments, I simply am I, an enduring, conscious presence given directly and pre-reflectively to awareness absent any analytic reckoning (e.g., Neuhouser, 1990; Kant, 1998; Klein, 2014a).

### The continuity of the subjective self: evidence-based diachronicity

In the section titled Sameness and the Self: The Problem of Personal Diachronicity I made the observation that the self of first-person subjectivity entails a feeling, and that this feeling does not vary over time. In that sense, it always is present as an “experiential given” underpinning our feeling of sameness (cf., James, 1890).

However, this is not to imply that this feeling serves as a *comparative* basis (i.e., with past feelings of sameness) thereby supporting a conclusion of temporal continuity. To do so would be to conflate the modes of operation of two ontologically distinct aspects of the self—the neuro-psychological (e.g., memory-based comparisons) with the subjective, non-evaluative aspect of self (my reasons for positing ontological separability—but causal relatedness—between these two aspects of the self are given in Klein (2014a). I cannot repeat them here, as to do so would greatly exceed the limits on word count for manuscripts of this type. Accordingly, the interested—or confused—reader is referred to arguments presented in detail in the above reference. I apologize in advance for any lack of clarity within the present text).

Moreover, to construe the felt invariance of the subjective self as a basis for comparative judgments of personal diachronicity would conflate evidential with felt sameness. These two modes of experiencing sameness, I am arguing, need to be kept both conceptually and functionally distinct.

However, for some this will seem to beg the question of why or how felt invariance translates into a directly given, conceptually unanalyzed sense of being a temporal continuant—a feeling that, under most circumstances, we take as default—i.e., it is an un-reflected core aspect of our experiential being—and thus does not require (and is not subjected) to critical analysis.

Two considerations merit mention. First, there is no reason why felt sameness cannot be taken as an object of subjectivity and consequently evaluated. Indeed, I suspect it often is when motivation (either internally or externally mandated) argues in favor of considerations of evidential support for personal diachronicity. Second, however, I also am arguing that evidential criteria typically are not part of our experience of continuity. Rather, what underwrites are feeling of being a personal continuant is just that—the pre-reflectively given, conceptually unexamined feeling that I am I (e.g., Zahavi, 1999; Strawson, 2005). In this sense, personal diachronicity is not even (typically) a belief (though it can become so under circumstances calling for evidential warrant); rather it is a background presumption that is as much a part of our phenomenology as is the feeling that “I am alive” (e.g., we simply take it as an un-reflected given absent any analysis—though reasons can be provided when necessary).

With these considerations in mind, let's turn again, the case of patient D.B., whose uninterrupted access to personal memory was severely restricted. Might his intact subjectivity provide a basis for his sense of diachronicity? The answer depends on the criteria we use to investigate one's sense of personal diachronicity and the manner in which “sense of personal diachronicity” is conceptualized.

Seen in terms of *evidential* criteria, episodic memory loss renders patients such as D.B. and K.C. (e.g., Tulving, 1993; Klein et al., 2002c) unable to access information about their life history—i.e., their lived past as well as imagined future (for a recent review, see Klein, 2013a). Patient K.C., who suffered a total loss of episodic memory due to a motorcycle accident, describes his personal future as content-free and informationally vacant (it is important to note that individuals with intact episodic memory have no problem imagining content-rich, future-oriented personal scenarios; for a recent review see Klein, 2013a)^9^.

E.T.: (Endel Tulving): “Let's try the question again about the future. What will you be doing tomorrow?”
K.C.: smiles faintly (following a 15-s pause) and responds: “I don't know.”
E.T.: “Do you remember the question?”
K.C.: “About what I will be doing tomorrow?”
E.T.: “Yes. How would you describe your state of mind when you try to think about it?”
K.C., after a 5-s pause, replies: “Blank, I guess.”

(Tulving, 1985; Tulving, p. 4: Note, in the original, patient K.C. was referred to as N.N.)

D.B. shows similar difficulties. When asked to provide information about personal events in his past, he is at a complete loss. In addition, he shows a conspicuous inability to project himself into an imagined future (Klein et al., 2002b).

These limitations in evidence-based mental time travel—and the difficulties they present for the sense of personal diachronicity construed as an evidence-based ability to subjectively navigate personal time—are not due to patients' difficulties comprehending the meaning of temporal concepts. Unpublished data (Klein, 2000; Craver, 2013) make plain that both K.C. and D.B. have a firm grasp of the concepts of past, present and future.

In response to the question “What is the future?” K.C. replies “Events that haven't happened yet,” while the question “What is the past?” is answered “Events that have already happened”. Asked “Can you change the past?” K.C. emphatically states “No!” When queried “Can you change the future, and if so, how?” he observes “Yes. By doing different things.” To the question “Can something that happens in the future change what has happened in the past?” K.C. again responds with an emphatic “No,” while the query “If an event is in the future will it always stay in the future?” elicits the response “No. Because time moves on.”

Patient D.B. also presents a nuanced understanding of temporality. In response to the question “What is the future?” he answers “Things that haven't happened yet, but someday will.” He describes the past as “Things that happened before… but are not happening now.” Asked “Can you change the past?” D.B. says: “Don't think so, unless you had a time machine or something. Don't think so… not really. Maybe in science fiction (laughs).” To the question “Can the past influence the present?” he replies “Sure. All the time… that's the way things work.”

In short, when the sense of personal diachronicity is conceptualized in terms of evidential criteria, individuals lacking total access to episodic memory (as well as suffering impairments of semantic personal knowledge) show a profound inability to engage in personally-relevant temporal extension. They are unable to (a) provide evidence-based knowledge of their personal past or (b) generate content-based personal future scenarios.

### The continuity of the subjective self: felt diachronicity

When personal diachronicity is considered in terms of *felt* rather than evidence-based criteria, however, a markedly different picture of personal continuity emerges. As we have seen, when he was asked to recall his past or to describe his possible future, D.B.'s interlocutors were met either with uncomfortable silence or expressed bewilderment.Gaping holes in his corpus of self-knowledge —brought to his attention by explicit requests—caused D.B. confusion, concern and fear; i.e., the type of reactions one would expect from a mentally coherent individual unable to fully comprehend the evidential vacuum experienced by his subjective self (Klein, 2012, 2014b).

This is a critical point, but one easily missed: When requested to provide evidence in support of his sense of personal diachronicity, D.B. expresses agitated concern: “I should, shouldn't I?” he wonders aloud. But he can't. In response to my query “Do you feel as though you are the same person you were before your heart attack?” D.B. replies: “If you mean, am I the same person… well not really. I have these head issues you know… can't seem to remember like I use to. But if you mean have I, D.B. (for confidentiality, this is not the name he actually used), lived a long life… well, of course. And I hope to keep at it.” In short, D.B. is troubled when made aware (either by personal concerns or the requests of others) of the unavailability of evidence that, under normal circumstances, would be available to inform his sense of self as a temporal continuant.

This clearly is *not* a person lacking a sense of temporal persistence (although he is unable martial evidence in support of that sense). He is concerned about the fate that has befallen his (apparently intact) feeling of himself as an enduring entity. What he lacks is the ability to supplement this feeling with evidential offerings from his material self. Interestingly, the absence of an ability to recollect a personal past or imagine a personal future does not appear either to trouble or to capture the attention of his subjective sense of self *unless* the situation makes his deficits the object of his awareness.

A similar appreciation of the continuity of self in the presence of evidential deficit is found with patient H.M. Replying to the question “How do you feel about yourself?” he observes “I feel I have failed more than the average person… I feel like a complete failure as a person… I am disappointed in myself.” (Hilts, 1995, p. 153). Like D.B., H.M. may not be able to offer evidential support for his feeling of continuity, but he clearly feels himself to be a temporal continuant, one whose past acts have failed to meet his current expectations. Apparently, something more than *evidential* criteria is at work in underwriting one's sense of diachronicity.

A particularly compelling example of intact sense of personal diachronicity in the presence of severe impairment to the evidential bases for that felt sameness comes from the case of Zasetsky (Luria, 1972). Zasetsky was a Russian soldier, who, as the result of battle, was left aphasic, perceptually and proprioceptively disoriented and hemianopic. He also became densely amnesic, with severe impairment (both antrograde and retrograde) of episodic as well as semantic memory function.

As a result of deficits in proprioception and kinesthetic feedback, Zasetsky had trouble feeling and locating parts of his own body. His perception of the external world suffered as well. External objects either were nonexistent or appeared as fragmented, flickering background entities.

Having lost most of his personal memory, his ability to recall his past and plan for his future was virtually non-existent (for a recent discussion of the relation between memory and mental time travel, see Klein, 2013a). He professed to have no clear idea of his preferences, beliefs, values, or goals. In short, Zasetsky was unable to access most of his sources of epistemic self-knowledge.

Despite the great challenges presented, Zasetsky struggled to piece together the evidential fragments that remained from his material self. Under the patient tutelage of Luria and others, he slowly and painfully regained some rudimentary ability to read, write and perform basic bodily functions. As a consequence, he was able to provide Luria with a record of his thoughts and feelings about the changes to the self brought about by his difficulty providing first-person subjectivity with content from the now largely dysfunctional aspects of his material self.

But- and this is the key point—despite monumental loss of access to material bases of self, Zasetsky maintained a feeling of personal sameness. *He* was painfully aware of *his* deficits and greatly troubled by their effects on *his* ability to place *himself* physically, temporally and spatially. *He* complained often about the confusion engendered by impairments of perceptual, kinesthetic and proprioceptive feedback; *he* was disorientated by *his* loss of preferences and difficulties imagining *his* future or recalling *his* past.

Thus, at no time was his subjective self-awareness lost (save, perhaps, periods of dreamless sleep): The “I” always was there—troubled, bewildered, angered, and confused by its loss of access to sources of self-knowledge, yet determined to salvage whatever it could of a life left in cognitive and perceptual shambles. In the end, it was this subjectively felt determination to improve his situation that led Zasetsky to undertake the arduous rehabilitative program that enabled the subjective self to regain partial contact with the external world and aspects of the material self. He doggedly maintained hope for a life better than the one that had befallen him in battle. And “hope” is word whose meaning unambiguously implies a sense of self as a personal continuant.

In short, there is strong empirical support for the proposition that a person, absent most of what we would place under the heading of “material self” still can retain a clear feeling of his or her sameness and temporal continuity. What is particularly noteworthy in Zaztesky's case are his concerted efforts to distance himself from what he had become and recapture a semblance of normality.

### The timelessness of the subjective self

One fascinating, but often overlooked, aspect of the experience of patients with temporally graded amnesia (e.g., the law of “first in, last out”; Ribot, 1882) is that the subjective aspect of self typically is not confused by, or troubled over, its inability to recollect events and experiences covered by memory loss (unless, of course, the self is confronted with evidence of the incongruity between the passage of time and current self-beliefs. Absent such confrontation, the patient appears relatively content to see him or herself as being of the age at which personal memories remains available; for review and discussion, see Klein, 2012).

Consider, for example, the case of patient, J.G. (Sacks, 1985). As the result of Korsakoff syndrome, J.G. was unable to recollect any personal happenings postdating 1948. Despite passage of nearly 30 years since the onset of his amnesia, testing revealed that J.G. believes he still is a young man, and that the year still is 1948 (it was 1975). Consistent with his beliefs, on being shown his face in a mirror (i.e., that of a much older man) J.G. is stunned and confused. Fortunately, due to his amnesia, after a few moments of distraction and J.G. once again is relaxed and comfortably situated in 1948.

The remarkable case of patient B. (Storring, 1936) brings the relation between memory, personal temporality and the subjective self into strong relief. As a result of a gas poisoning accident, patient B. was rendered incapable of remembering anything occurring post-injury for more than roughly one second! At the time of testing (the mid-1930's) he knew nothing of the life he had lived post-poisoning or of his marriage of the past 5 years. Like J.G., he is perplexed every time he sees himself in a mirror – 10 years earlier he looked much different. While psycho-physcial aspects of his self have changed with time, the subjective aspect of self-shows no comparable evidence of change: For B.'s first-person subjectivity, it is, and always will be May 1926.

There are many aspects of this case that merit extensive discussion. For my purposes, however, the relevant features pertain to what it can tell us about B.'s self of first-person subjectivity, a self whose knowledge of the aging process has been decoupled from changes to the material self-brought about by the passage of time. The subjective self, no longer having access to these changes, does not show a parallel aging of its own. B. has become a man of the eternal present.

However, as Storring (1936) notes at length, B. is *not* a man of the moment: “B. gives meaning to the situation before his senses. And it is this context that reaches from one second to the next that creates the flowing transition. A sensible, reasonable task is harmoniously carried to its completion, regardless of how long it takes, because … the rational whole is known in the situation as a goal which is then fulfilled” (Storring, 1936, pp. 75–76). This is a person, Storring concludes, with a second-long consciousness that nevertheless has a clear sense of personal continuity. The subjective self, anchored in the past as a result of disruption of sensory and cognitive processes, nevertheless, remains a constant, experiencing, feeling, thinking center of subjectivity unperturbed by the passage of time.

The take-away message is that seldom, if ever, do we find a patient who claims to experience himself as much older than his or her intact recollections would suggest; rather, we find the reverse—the patient resides in the past (provided he or she has access to some personal recollections) and is troubled only when a discrepancy between content provided by the material self (or one's senses) fails to match current beliefs (Klein, 2012). The self of first-person subjectivity thus seems outside of the aging process, accepting whatever the material self has to offer vis a vis evidence of temporal placement.

## Personal diachronicity and the sense of self

At this point in the discussion, a reasonable question concerns the extent to which personal diachronicity is a “phenomenological given.” That is, to what degree are considerations of self-continuity the result of social requests, moral obligations and conceptual curiosity, as opposed to a basic attitude we spontaneously adopt toward our everyday experience of self?

If one accepts the proposition that first-person subjectivity is a pre-reflectively given and ageless aspect of the subjective self, then diachronicity *per se* may not actually be a live issue for one's everyday sense of self. It may only become so when a person attempts to provide *evidential* support for personal continuance (e.g., from memories supplied by the material aspect of self). On this view, diachronicity is not so much felt as it is logically constructed from informational content (which, as we have seen, does not easily translate into unambiguous criteria for personal continuity).

Personal diachronicity thus may come into play largely at the evidential level. Since, the self of first-person subjectivity is not experienced as changing with time, considerations of personal diachronicity typically are not a part of our sense of self. They become a part when the self is taken as an object of reflection; and this, in turn, occurs when we are called on—either by personal concerns or external contingencies—to directly address issues pertaining to the self as a temporal continuant.

An obvious objection would be that “the reason that the self of subjectivity in not experienced as changing is because the self of subjectivity is not experienced at all.” While such an objection has some force, I believe there are two different responses that can partly address this concern. First, first-person subjectivity is perhaps the single most salient aspect of everyday experience. Of course, it is always (per Brentano) conflated with intentional objects (although advocates of “pure consciousness” argue that subjective states absent intentional objects can, with extensive training, be attained; e.g., Forman, 1990). But, as many have argued (for recent reviews, see Legrand and Ruby, 2009; Klein, 2012, 2014a), our acquaintance with the self of first-person subjectivity is a necessary postulate to capture what we mean by a sense of oneself. Second, the evidence presented in this paper of patient's suffering varying degrees of cognitive impairment, yet still maintaining a coherent sense of diachronicity are consistent with the notion that what underwrites this feeling is the constancy of subjectivity and not the flickering or non-existent objects taken by that subjectivity. While these arguments are consistent with the assumption that the felt invariance of subjectivity underlies the sense of sameness, it must be acknowledged that neither provides a conclusive refutation of the objection raised. Accordingly, it remains a live possibility.

In summary, we typically do not *feel* ourselves to be different over time. When we do, it most often is the result of our continuity being called to question by self or other. Moreover, to the extent that memory, in particular, and evolutionary considerations, in general, play a part in our sense of temporal continuity, it is the “now and the next”, not the past, to which our pre-reflective sentiments gravitate (for discussion of the future-orientation of memory, see Klein, 2013b). As Strawson (personal communication) puts it, the temporality of subjective self consists in “and now and now and now.”^10^

We should not draw from these observations the conclusion that the subjective aspect of self necessarily is immortal or transcendental. It very well may be incapable of existing apart from the body (e.g., Olson, 2007). It may be an emergent property of the material self (e.g., Hasker, 1999). But this emergence—if indeed it is emergence—is something we clearly do not know how to deal with within the context of current theory and research in science and philosophy.

We are a long way from beginning to answer questions about the self of first-person subjectivity. Yet, in my opinion, answers to these questions are fundamental for a psychology that takes as its goal the full appreciation of human experience.

## Some final thoughts

In this paper, I have argued that our non-analytic, pre-reflective feeling of the self may be the primary determinant of our intuition of self as temporally extended. Explicit considerations of personal diachronicity come into play primarily when contingencies make it necessary to contemplate (and provide evidence in support of) personal continuity. In their absence, one's sense of self as a temporal continuant is more one of unreflected acceptance than explicit formulation. Sameness is the self's default mode – a felt identity uninformed by evidence. And, in virtue of the unchanging nature of subjectivity, diachronicity becomes a concern of the self only when considerations of personal temporality are selected by environmental demand, personal concern or philosophical query to serve as the objects of subjectivity^11^.

Plato maintained that true knowledge could only be sensed by the soul. Aristotle, in contrast, believed knowledge is derived from evidence provided by the body. The tension between sense and evidence has been a source of academic, social, political and religious debate (often acrimonious) for more than two millennia, with the emphasis shifting as a function of cultural as well as intellectual imperatives (e.g., Koestler, 1989).

In this paper I have made my case for the non-evidential basis of one's sense of sameness over time. However, this should not be seen as a call to reaffirm the Platonic distaste of understanding by reliance on the “grossness of bodily senses.” Rather, it is an appeal to broaden our criteria for understanding beyond the reductionist materialism that characterizes much of Western thinking, and to embrace the possibility that there are aspects of reality that may not (easily) submit to such highly circumscribed treatment (e.g., Meixner, 2008; Papa-Grimaldi, 2010; Nagel, 2012; Klein, 2014a). Some phenomena, if they are to be saved, can be saved only if we allow that knowledge by acquaintance (e.g., Russell, 1912/1999) sometimes may be the metaphysically propitious stance. Personal diachronicity very well may be a case in point.

An appreciation of “reality” in its fullness likely requires we strike a balance between the different approaches to knowledge championed by Plato and Aristotle. Only by affecting a rapprochement between these “seemingly” conflicting metaphysical commitments will a sufficiently inclusive understanding of reality be a potentially realizable objective.

### Conflict of interest statement

The author declares that the research was conducted in the absence of any commercial or financial relationships that could be construed as a potential conflict of interest.

## Footnotes

^1^This is not to suggest that questions pertaining to less demanding notions of identity are ignored by philosophers. This is far from the case (e.g., Wiggins, 1971; Brennan, 1988; Noonan, 1989; Gallios, 1998). For example, questions such as absolute vs. relative identity, the Ship of Theseus paradox and the identity of a clay sculpture and the unformed lump out of which it was fashioned are some of the less exacting, boundary issues of identity debated by philosophers.

^2^In this paper, I sometimes will use the term “person” in place of the term “self”. This philosophically debatable move is one not everyone will be comfortable with (e.g., Locke, 1689–1700/1975; Wilkes, 1988). My (occasional) substitution of terms entails nothing beyond expositional convenience. While I recognize the conceptual issues it raises, there should be little question of the meaning I intend.

^3^Questions pertaining to how a subject takes itself qua subject, to be one and the same, open the door to complex issues of self-reflexivity and the philosophical puzzles they engender (e.g., Falk, 1995; Bolander et al., 2006; Strawson, 2009). Their treatment is beyond the scope of this paper.

^4^In Klein (2012, 2014a) I use the terms “epistemological self” and “ontological self” to describe the “material self” and the “self of first-person subjectivity,” respectively. My reasons for this unconventional usage are complex (Klein, 2014a) and need not concern us here. For the clarity that comes with conceptual familiarity, the latter terminology (i.e., the material and subjective aspects of self) are adopted in the present text.

^5^Rapid and substantial change, by contrast, can lead to serious doubts about personal continuity. The classic case of Phineas Gage, who suffered profound changes in personality closely following brain injury, led to the well-known observation by his attending physician that Gage was “no longer Gage.” (e.g., O'Driscoll and Leach, 1998).

^6^To argue that episodic memory is neither necessary nor sufficient, one would need to produce a case in which a person has episodic memory but no semantic memory, and that under these circumstances a sense of personal diachronicity was absent. Such a case, however, is not found in the annals of neuroscience (and I am not sure that a situation in which a person has intact episodic memory accompanied by complete absence of semantic memory is—on definitional, linguistic or phylogenetic grounds—possible).

^7^The relation between semantic trait self-knowledge and episodic recollections of trait-relevant behavior is a complicated affair. Suffice it to say that a substantial body of research shows that the respective roles of these two systems of memory in the creation of trait self-knowledge depend on a large number of factors (for a recent review see Klein et al., 2008).

^8^For example, it should require time to reconstruct a coherent, “sufficiently” unbroken self-narrative; thus, evidential sources of diachronicity could not easily provide the immediate sense that I am the continuing existent I take it most people refer to when they claim to experience sameness of self over time.

^9^Future-oriented mental time travel is well-known to depend on memory (for recent reviews see Szpunar, 2010; Schacter, 2012; Klein, 2013a).

^10^The Earl of Shaftesbury (1698) captures in a few well-chosen sentences much of what I have been struggling to say: “The metaphysicians… affirm that if memory be taken away, the self is lost. [But] what matter for memory? What have I to do with that part? If, whilst I am, I am as I should be, what do I care more? And thus let me lose self every hour, and be 20 successive selfs, or new selfs,' tis all one to me: so [long as] I lose not my opinion [i.e., my overall outlook, my character, my moral identity]. If I carry that with me'tis I; all is well… —The now; the now. Mind this: in this is all.” (cited in Strawson, 2008, p. 198, parenthetical comments added).

^11^Ricoeur's (1994) distinction between sameness (memete) and selfhood (ipseite) is particularly germane to our discussion of personal diachronicity. Ricoeur argues that selfhood is maintained despite changes in the evidential criteria for sameness (i.e., character dispositions and other marks that permit the reindentification of an individual as being the same over time). This view, based exclusively on philosophical considerations, offers strong support for our empirical observations concerning the insufficiency of evidential criteria for continuity of self.
